# Genipin-Crosslinked, Proteosaccharide Scaffolds for Potential Neural Tissue Engineering Applications

**DOI:** 10.3390/pharmaceutics14020441

**Published:** 2022-02-18

**Authors:** Henna Cassimjee, Pradeep Kumar, Philemon Ubanako, Yahya E. Choonara

**Affiliations:** Wits Advanced Drug Delivery Platform Research Unit, Department of Pharmacy and Pharmacology, School of Therapeutic Sciences, Faculty of Health Sciences, University of the Witwatersrand, Johannesburg 2193, South Africa; 1054426@students.wits.ac.za (H.C.); pradeep.kumar@wits.ac.za (P.K.); philemon.ubanako@wits.ac.za (P.U.)

**Keywords:** chitosan, hyaluronic acid, neural, extracellular matrix, proteosaccharide, gelatin, crosslinking, genipin, dexamethasone, drug release, scaffold

## Abstract

Traumatic brain injuries (TBIs) are still a challenge for the field of modern medicine. Many treatment options such as autologous grafts and stem cells show limited promise for the treatment and the reversibility of damage caused by TBIs. Injury beyond the critical size necessitates the implementation of scaffolds that function as surrogate extracellular matrices. Two scaffolds were synthesised utilising polysaccharides, chitosan and hyaluronic acid in conjunction with gelatin. Both scaffolds were chemically crosslinked using a naturally derived crosslinker, Genipin. The polysaccharides increased the mechanical strength of each scaffold, while gelatin provided the bioactive sequence, which promoted cellular interactions. The effect of crosslinking was investigated, and the crosslinked hydrogels showed higher thermal decomposition temperatures, increased resistance to degradation, and pore sizes ranging from 72.789 ± 16.85 µm for the full interpenetrating polymer networks (IPNs) and 84.289 ± 7.658 μm for the semi-IPN. The scaffolds were loaded with Dexamethasone-21-phosphate to investigate their efficacy as a drug delivery vehicle, and the full IPN showed a 100% release in 10 days, while the semi-IPN showed a burst release in 6 h. Both scaffolds stimulated the proliferation of rat pheochromocytoma (PC12) and human glioblastoma multiforme (A172) cell cultures and also provided signals for A172 cell migration. Both scaffolds can be used as potential drug delivery vehicles and as artificial extracellular matrices for potential neural regeneration.

## 1. Introduction

Tissue engineering aims to address the continuous rise in the need for organ transplants by providing a suitable surrogate matrix that promotes the regeneration of damaged tissues. Research in this field is aimed at the use of naturally occurring polymers that are bioactive, biocompatible and non-toxic. The ultimate objective of tissue engineering is to create duplicates of native tissue, which can function as substitutes and promote regeneration. The creation of three-dimensional (3D) scaffolds is crucial in the field of tissue engineering. These scaffolds not only function as templates upon which new tissue can be produced but also need to be porous to ensure adequate transport of oxygen and nutrients [1]. Mechanical properties such as compressive strength and stiffness also dictate the success of a scaffold at its site of action, specifically in the regeneration of soft tissue such as cartilage. The scaffolds can be produced from natural or synthetic polymers, with the former being favoured for their low toxicity.

The use of polysaccharides such as chitosan and hyaluronic acid (HA) in regenerative medicine has become increasingly popular. Chitosan is a semi-crystalline polysaccharide obtained by the deacetylation of chitin and is a co-polymer of d-glucosamine and N-acetyl d- glucosamine. Chitosan is insoluble in solutions above pH of 7; however, in weakly acidic solutions (pH 6), the free amine groups of chitosan are protonated, which allows the molecule to be solubilised. Chitosan has been used extensively due to its biocompatibility, hydrophilicity, biodegradability and structural similarity to glycosaminoglycans (GAGs) found in the extracellular matrix (ECM). However, chitosan does not possess significant mechanical strength. This can be overcome by diverse methods of crosslinking, such as photo-crosslinking and chemical crosslinking. Its cationic nature allows for electrostatic interactions with other polymers. Previous studies have shown that chitosan supports the attachment and proliferation of fibroblasts, chondrocytes, neurites and hepatocytes, thus making it a promising candidate for tissue engineering applications. Chitosan has been employed in the field of neural tissue engineering due to it being an effective neuroprotector following spinal cord injuries [2]. Mo and researchers (2010) observed the migration of cells and axonal stimulation when a chitosan scaffold was implanted into the rat hippocampus. This led to re-established continuity of the neural circuit, with an improved cognitive capacity [3]. Other advantages include its biocompatibility, biodegradability, antibacterial activity and low toxicity [4,5,6,7].

Gelatin, which is obtained by the hydrolysis of collagen and is a main component of the ECM, has also been widely used due to its antigenicity and biocompatibility [8]. Hydrolysis is of collagen results in the breakage of its triple helical structure, which yields gelatin [9]. Gelatin is an anionic protein, thus favouring the formation of a polyelectrolyte complex with chitosan [10]. The blending of gelatin with chitosan furthermore improves the biological activity of the resulting scaffold as gelatin confers its Arg–Gly–Asp (RGD)-like sequence to the product, thus improving cell adhesion and proliferation [11]. Gelatin has been used in this study to reduce the stiffness of the hydrogel to match the mechanical strength of brain tissue. Furthermore, gelatin-rich constructs support the prolonged proliferation of stem cells and multiple neurons along with their plasticity [12]. Gelatin, however, dissolves rapidly in an aqueous environment, thus necessitating crosslinking [13]. Physical crosslinking of these polymers is advantageous, as they do not cause potential harm; however, the desired degree of crosslinking can be difficult to obtain. Thus, chemical crosslinkers such as glutaraldehyde, carbodiimides and genipin can be used [14].

Until today, chitosan–gelatin composites have been used extensively in tissue engineering, notably in hepatic, skin and cartilage regeneration [15,16]. The blends are beneficial due to their porosity, biocompatibility and mechanical properties. The blend is, however, unstable in aqueous solutions and readily undergoes dissolution without the presence of crosslinkers.

Hyaluronic acid (HA) is an anionic polysaccharide that can be found in the ECM of the brain, cartilage and epithelial tissue [6,17,18]. HA functions through various receptors in the brain, mainly CD44 and the hyaluronan associated mobility receptor (RHAMM), and plays an extensive role in cellular interactions such as migration and proliferation, which impact haemostasis in the brain [19]. The molecular weight of HA influences cellular responses, with low molecular weight HA (80–800 kDa) inducing the production of pro-inflammatory markers and high Mw (>1600 kDa) inhibiting these mediators [20]. Other researchers have shown that low Mw HA enhanced the proliferation of astrocytes in rat spinal cord injuries and high molecular weight reduced the proliferation of astrocytes [21]. HA can easily be modified through its three different functional groups, the glucuronic acid group, the primary and secondary hydroxyl groups, and the N-acetyl group [22]. These modifications influence the mechanical properties and biological properties of HA; however, modification must be carried out carefully as a high degree of modification can impair its biological activity.

The main disadvantage of HA is the lack of cell adherence to its surface. This can be overcome by combining HA with other biologically active molecules. Spector and co-workers showed that the addition of collagen favoured neuronal differentiation [23,24]. The combination of gelatin and hyaluronic acid is not new to regenerative medicine and has been applied in cardiac, adipose and epithelial cell regeneration. In this study, a semi-interpenetrating polymer network (IPN) is prepared by crosslinking gelatin with genipin and blending it with HA to form a proteosaccharide scaffold. This is evaluated for its applications in TBIs and neural tissue engineering. High Mw HA is used in this study due to its stiffness and astrocyte inhibition activity.

Over the years, Genipin, which is derived from *Gardenia Jasminoides*, has replaced glutaraldehyde and other carbodiimide crosslinkers due to the expanded biochemical significance of genipin-crosslinked hydrogels but also due to its stability, biocompatibility and predictable chemistry [25]. Cytotoxicity studies of genipin against fibroblasts showed that genipin was 10,000-fold less toxic than glutaraledehyde, and fibroblasts proliferated 5000-fold more as compared to when treated with glutaraldehyde [26]. Da Silva and co-workers (2021) showed that the genipin component of the IPNs researched exhibited antimicrobial properties against nine bacterial pathogens associated with postsurgical brain infections, which justifies the use of genipin in neural scaffolds [23]. In this research, genipin crosslinked chitosan–gelatin and HA–gelatin scaffolds are prepared and evaluated for their application in neural tissue engineering, an area of regenerative medicine that it has not previously been applied in. These proteosaccharides overcome the limitations associated with the use of their pristine forms. Due to gelatin possessing weak mechanical strength, it is used in combination with hyaluronic acid, which, due to its stiffness in a hydrated form, imparts mechanical integrity to the scaffolds. In addition, HA provides good hydration and diffusivity of nutrients and waste compounds in vivo. Gelatin provides a peptide sequence to each proteosaccharide, which would be recognised by cell integrins and equip the proteosaccharides with bioactivity.

## 2. Materials and Methods

Low molecular weight chitosan, Gelatin (Type B) and glacial acetic acid were purchased from Sigma-Aldrich (St. Louis, MO, USA). Genipin was purchased from Challenge Bioproducts Co., Ltd. (Yun-Lin Hsien, Taiwan). HA from Streptococcus Equi was purchased from Sigma-Aldrich (St. Louis, MO, USA). Dulbecco’s Modified Eagles Medium-high glucose, Roche Cell Proliferation Kit I (MTT), dimethyl sulfoxide (DMSO) and trypsin–EDTA solution were obtained from Sigma Aldrich (Steinheim, Germany). Donor Equine Serum (DES) was purchased from Hyclone (Logan, UT, USA). Ethanol (99% absolute) was purchased from LabChem, (Johannesburg, South Africa). Fetal Bovine Serum and Ham’s F-12 Nutrient Mix (F12) were purchased from (PAN Biotech, Aidenbach, Germany). Rat (Rattus Norvegicus) adrenal gland pheochromocytoma (PC12) and human glioblastoma multiforme (A172) cells were purchased from Cellonex (Separations, South Africa). Penicillin/Streptomycin/Amphotericin B (P/S/AB) solution was purchased from Lonza (Bend, OR, USA).

### 2.1. Synthesis of the Full IPN and Semi-IPN

**Figure 1:**
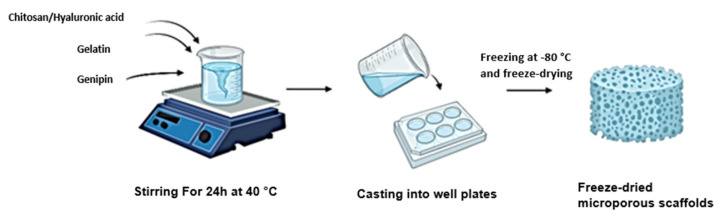
Schematic showing the synthesis of each scaffold through a mixing crosslinking method.

A 2% solution of chitosan was prepared by dissolving 2 g in 100 mL of 0.5 M glacial acetic acid. A 2% solution of gelatin was prepared by dissolving 2 g in 100 mL of distilled water over a magnetic stirrer at 50°c. These solutions were blended and subsequently crosslinked with a 0.01% *w*/*v* solution of genipin, obtained by dissolving 100 mg in a 1:4 ethanol–distilled water solution. A mixing crosslinking method was used to prepare the hydrogels. A semi-IPN was prepared similarly by substituting chitosan with HA dissolved in cold distilled water. An optimised 50:50 volumetric ratio of protein to polysaccharide was used in the preparation of the semi-IPN and full IPN. Genipin was added dropwise to each blend at a concentration of 1% *v*/*v*. The blends were allowed to crosslink for a period of 24 h until a blue pigment had appeared, and the gel showed an increased viscosity, which indicated the reaction of genipin with amino groups present in each blend. A volume of 250 µL of each hydrogel was cast into each well of a 24-well plate and was subsequently frozen at −80 °C and freeze-dried in a lyophiliser for 14 h. Porous scaffolds were obtained and subsequently washed with ethanol to remove excess crosslinker (Figure 1).

### 2.2. Attenuated Total Reflection Fourier Transform Infrared Spectroscopy (ATR-FTIR) of Pristine Polymers and Their Crosslinked Networks

Scaffolds were analysed with a Spectrum 2000 FTIR Spectrometer (PerkinElmer, Massachusetts, USA) at wave numbers of 4000–650 cm^−1^ at 10 scans/second under a constant pressure of 120 psi and a single-reflection diamond MIRTGS detector. The stage for the sample was cleaned with methanol prior to analysis, and a background spectrum was obtained. The spectra obtained were plotted on OriginPro, and the formation of amide bonds was confirmed in the region 1600–1700 cm^−1^.

### 2.3. Rheological Analysis of the Proteosaccharide Hydrogels

The Elastosens BIO2 (Rheolution Instruments, Montreal, Canada) was used to analyse the viscoelastic behaviour of the resulting networks. A laser was used to measure the displacement of the polymers to minimum amplitude vibration. A volume of 2 mL of each proteosaccharide hydrogel was added to a sample receptacle, which encapsulated an elastic membrane. This was done immediately after the addition of genipin to determine the time taken for crosslinking to take place by monitoring the change in storage (G′) and loss modulus (G″). Viscoelastic behaviour of each hydrogel was observed by tilting each bottle containing the hydrogels. The formation of a meniscus indicated the absence of gelation, while the absence of a meniscus upon tilting indicated hydrogel formation. A tilt bottle test was used for visual confirmation of crosslinking. The gels were photographed after 24 h of crosslinking to show the flow and crosslinking intensity of each hydrogel prior to lyophilisation.

### 2.4. Analysis of Crystallinity of the Proteosaccharide Scaffolds

X-ray diffraction using the Rigaku Miniflex 60 (Rigaku Corporation, Tokyo, Japan) was used to determine the degree of crystallinity of the scaffold matrix. Each scaffold was mounted using carbon tape and flattened with a glass slip. The proteosaccharide matrix was scanned at a start angle of 5° and a stop angle of 60° at 10°/minute over a 2θ range. This was conducted at room temperature. The crystallinity index was calculated using the following equation:

(1) $$Crystallinity\;Index\;\left(Ci\right)=\frac{Sum\;of\;areas\;under\;crystalline\;peaks}{Total\;area\;under\;crystalline\;and\;amorphous\;peaks}$$

### 2.5. Thermoanalytical Analysis of the Lyophilised Proteosaccharide Scaffolds

Proteosaccharide-based scaffolds were analysed using the Mettler Toledo, DSC1, STARe System (Schwerzenback, Switzerland). The scaffolds weighing 5–10 mg were heated at a temperature range of 10–300 °C at 10 °C /minute to identify degradation, melting point, and the glass transition temperature (Tg) in an aluminium pan (40 DL) and sealed with a 0.2 mm opening in the lid for heat flow. This range was used to establish the temperature at which the proteosaccharides will degrade. The test was run under constant N_2_ gas. The resulting thermogram was compared to a reference thermogram run with an empty sample holder.

### 2.6. Swelling Potential of Scaffolds under Physiological Conditions

Swelling potential of the scaffolds was measured after immersing the scaffolds into PBS (pH = 7.4) until they had reached their equilibrium swelling after 2 h at 37 °C. Thereafter, the surface water was removed using filter paper, and samples were weighed (Wet weight). Experiments were done in triplicate. The swelling ratio was calculated using the following formula:

*Swelling ratio = (Wet Weight − Dry weight) / Dry weight* (2)

### 2.7. Morphological Analysis of Proteosaccharide Scaffolds

The morphological structure of a scaffold is critical to its function in tissue engineering. The scaffolds were observed under a scanning electron microscope (SEM) (FEI Nova NanoLab SEM, FEI Co., Hillsboro, OR, USA). Each scaffold was mounted on a stub using carbon tape and coated with carbon and a mixture of gold and palladium (Au/Pd). The structure and porosity were observed.

### 2.8. Determination of Bulk Density of Each Scaffold

The bulk density of each scaffold was determined using the following equation:

(3) $$\rho \mathrm{bulk}=\frac{mass}{volume}=\frac{4m}{\pi d^{2}h}$$

where ρ is the apparent density of porous material (g/mm^2^), m is the mass of scaffolds after saturation with water (gm), d is the diameter of scaffold formulations after saturation with water (cm), and *h* is the height of scaffolds after saturation with water (mm).

### 2.9. Degradation of Proteosaccharide Scaffolds

The rate of degradation of the scaffolds was measured by placing each scaffold into polytops consisting of phosphate-buffered saline and lysozyme (10,000 IU/mL) into an orbital shaker at 37 °C and 25 rpm. The degradation medium was changed every second day to mimic physiological sink conditions. Scaffolds were removed on days 0, 3, 7, 14, 21 and 28 and weighed after drying. The weight at each time interval was compared to the initial weight of the scaffolds, and the rate of degradation was determined. The degradation behaviour of a scaffold is crucial in preventing a premature collapse of the scaffold and the accumulation of potentially toxic by-products.

### 2.10. Drug Release of Dexamethasone-21-Phosphate from Proteosaccharide Scaffolds

Dexamethasone-21-phosphate (DEX) was loaded into hydrogels at a concentration of 0.25 mg/mL to compare drug release kinetics from the full IPN and semi-IPN hydrogel. Each scaffold of the same weight was immersed in 3 mL PBS (pH = 7.4) in an orbital shaker maintained at 37.5 °C. A volume of 2 mL was withdrawn from each sample and replaced with 2 mL of fresh PBS at different time intervals (t = 0.5 h, 1 h, 2 h, 4 h, 6 h, 8 h, 10 h, 12 h, 24 h and every 24 h thereafter). The absorbance was read at 242 nm. These were plotted and compared to a standard curve of DEX, and cumulative drug release was measured.

### 2.11. In Vitro Cytotoxicity Evaluation of the Full IPN and Semi-IPN

An XTT assay was performed to determine the cytotoxicity of the full IPN and semi-IPN formulations on rat pheochromocytoma cells isolated from rat adrenal medulla (PC12) and human glioblastoma cells (A172). The assay was only carried out on the full IPN and semi IPN conjugates and compared to proteosaccharide blends. The PC12 cells were cultured to 80% confluence in Ham’s F12 growth medium, supplemented with 2.5% fetal bovine serum (FBS), 15% donor equine serum (DES) and 1% (*v*/*v*) penicillin/streptomycin. The cells were cultured for 4 days in a T-75 flask to about 80% confluence with the medium replaced after washing with phosphate-buffered saline (PBS) every second day to remove cellular debris.

A172 cells were cultured in Dulbecco’s Modified Eagle’s Medium (DMEM), supplemented with 10% fetal bovine serum (FBS) and 1% penicillin–streptomycin in a T-25 culture flask in an incubator set at 37°c and 5% CO_2_. For seeding, cells were detached using 0.25% trypsin and neutralised by adding 1 mL of media. The cell suspension was then aspirated and centrifuged at 1500 rpm for five minutes, and the cell pellet was resuspended in a fresh, supplemented growth medium. The trypan blue dye exclusion method was used to quantify viable cells in a hemacytometer, visualised on an inverted light microscope. Cells were seeded at a density of 3 × 10^4^ cells/mL and a volume of 90 µL per well in a 96-well plate.

The cells were incubated overnight, and 10 µL of IPN treatments was added to each well. Post-treatment, further cell incubation was done for 48 and 72 h at 37 °C. Thereafter, 50 µL of the XTT mixture (0.5 mL electron coupling reagent and 5 mL of XTT reagent) was added to each well, and the plates were incubated for 4 h at 37 °C. Absorbances were read on a Victor X3 microplate reader (Perkin Elmer, MA, USA) at 540 nm and 690 nm. The average absorbances were compared to that of the untreated and the positive control (10 µg/mL of 5-fluorouracil (5-FU)). The % cell viability was calculated as follows:

(4) $$\%\;cell\;viability=\frac{Average\;absorbance\;of\;treated\;wells-blank}{Average\;absorbance\;of\;untreated\;wells}\;\times \;100$$

### 2.12. Migration of A172 Cells in Response to the Semi- and Full IPN

A wound-healing assay was used to determine the rate of migration and wound closure of A172 cells. Cells were cultured in DMEM, supplemented with 10% FBS and 1% penicillin–streptomycin. The cells were cultured to full confluence at 37 °C, 5% CO_2_ in a humidified incubator. The cells were trypsinised, and viable cells were quantified using the trypan blue dye exclusion method, which made use of a trypan blue dye, a haemocytometer and an inverted light microscope (Olympus CKX53, Olympus, Tokyo Japan), using the 4× objective lens. After quantifying viable cells, the cells were seeded at a density of 6 × 10^4^ cells/mL in a 12-well plate. The cells were incubated for 48 h at 37 °C until a monolayer was established. A scratch was made in the centre of each well using a 200 µL micropipette tip, after which the wells were gently washed with PBS to remove dislodged cells and to achieve a clear-cut wound. IPN treatments were added at a 10% *v*/*v* concentration. The cells were observed over a period of 48 h, with images captured every 8 h using the phase-contrast feature and 4× objective lens of an inverted light microscope (Olympus CKX53, Olympus, Tokyo, Japan). The rate of wound closure/migration was calculated as follows:

(5) $$\%\;wound\;closure=\frac{Area\;of\;wound\;at\;specific\;time\;point}{Initial\;Area\;of\;wound}\;\times \;100$$

All images were captured using a phase-contrast microscope (Olympus CKX53, Tokyo, Japan) equipped with the LC micro software. Images were quantitatively analysed using ImageJ software.

### 2.13. Statistical Analysis

All experiments were carried out in triplicate, and statistical significance was determined by a student p test with *p* < 0.05 being statistically significant. Error bars were reported on all graphs.

## 3. Results

### 3.1. FTIR Analysis

**Figure 2:**
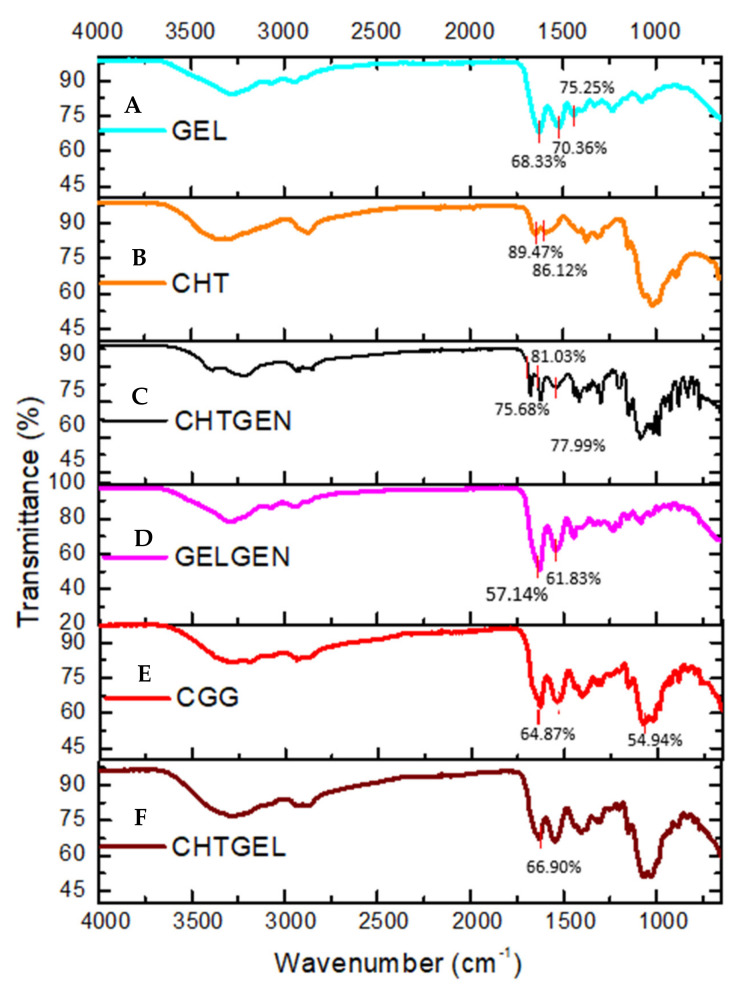
FTIR spectra of (**A**) pristine gelatin (GEL), (**B**) pristine chitosan (CHT), (**C**) chitosan-genipin (CHTGEN), (**D**) gelatin-genipin (GELGEN), (**E**) chitosan-gelatin-genipin (CGG) and (**F**) chitosan-gelatin (CHTGEL).

The FTIR spectrum of gelatin shows the typical N-H and O-H overlap at 3284.10 cm^−1^ while bands at 1627 cm^−1^, 1520 cm^−1^ and 1443 cm^−1^ are representative of the C=O stretching vibration of amide I, N-H bending vibration of amide II and carbonyl group, respectively (Figure 2A). In the FTIR spectrum of chitosan, the adsorption band at 3356.08 cm^−1^ indicates partially overlapping hydroxyl and amino groups (Figure 2B). Peaks between 2800 cm^−1^ represent C-H stretch vibrations. Peaks found at 1648.84 cm^−1^ and 1591.61 cm^−1^ represent the N-H bending vibrations of primary amine groups. Bands found at 1374 cm^−1^ are indicative of a C-O-H primary alcoholic group, while bands at 1023 cm^−1^ and 1149 cm^−1^ indicate C-O and C-N vibrations, respectively. FTIR of crosslinked chitosan shows the typical OH stretch at 3210.45 cm^−1^, with C-H vibrations at 2935.55 cm^−1^. The band found at 1535 cm^−1^ is indicative of the N-H deformation leading to the formation of a secondary amide due to the reaction of ester and O-H groups of genipin with the amino group of chitosan. The band found at 1623 cm^−1^ represents the C=O vibration of secondary amides. An increase in the number of bands between 1400 and 1000 cm^−1^, indicating C-N stretch and C-O-H vibrations, is observed after genipin crosslinking (Figure 2C). Characteristic peaks of gelatin are found at 3289.65 cm^−1^ and 2936.28 cm^−1^; however, due to the reaction of gelatin with genipin, the band at 1538.19 cm^−1^ is of greater intensity than in uncrosslinked gelatin due to the formation of C-N bonds, indicating amide bond formation (Figure 2D). FTIR spectrum of the CGG IPN network shows an increase in the intensity of band rations between 1629 cm^−1^ and 1538 cm^−1^. The band at 1538 cm^−1^ indicates the formation of C-N bonds due to the formation of a secondary amide as a result of the reaction between primary amino groups of chitosan and gelatin with the ester group of genipin. The band at 1064 cm^−1^ shows increased adsorption at the expense of the band at 1023 cm^−1^ of chitosan (Figure 2E). FTIR of the proteosaccharide hydrogel devoid of crosslinker shows the typical N-H and O-H stretch vibration at 3279.38 cm^−1^, and CH stretch at 2875 cm^−1^ of the spectrum shows no new bands; however, the increase in the intensity of the carbonyl band at 1629.13 cm^−1^ is indicative of proteosaccharide interaction (Figure 2F).

**Figure 3:**
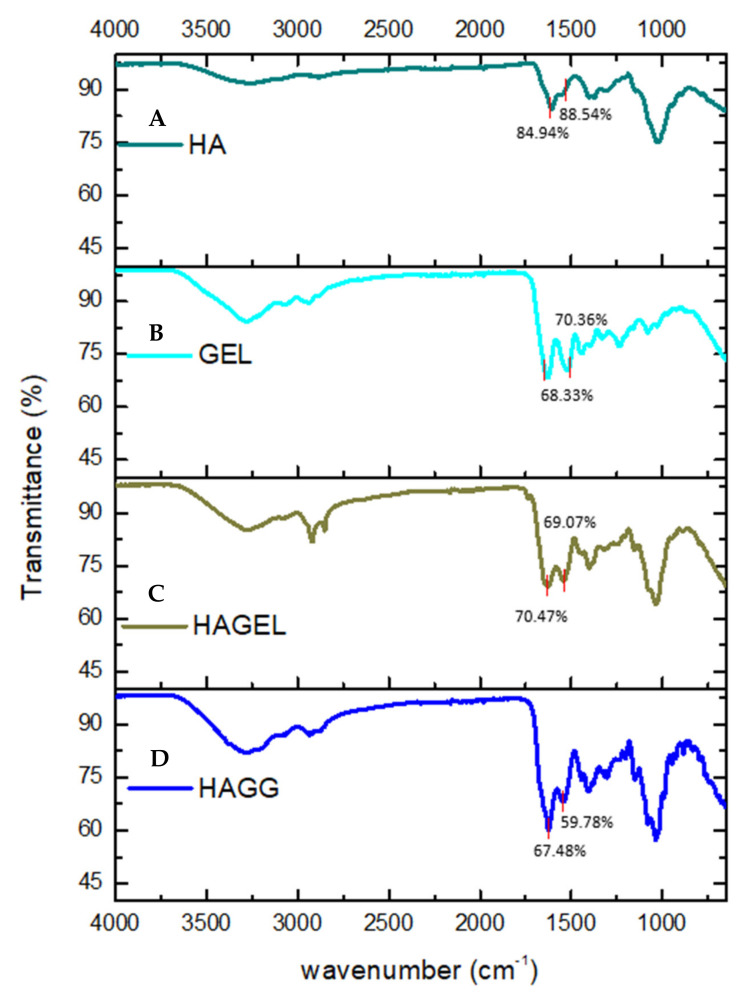
FTIR spectra of (**A**) pristine hyaluronic acid (HA), (**B**) pristine gelatin (GEL), (**C**) hyaluronic acid-gelatin (HAGEL) and (**D**) hyaluronic acid-gelatin–genipin (HAGG).

The FTIR spectrum of HA shows a vibration at 3254.81 cm^−1^, which is indicative of intra- and intermolecular OH stretch vibrations. The band found at 2872 cm^−1^ represents the CH_2_ group of HA. Bands at 1606.57 cm^−1^ and 1375.18 cm^−1^ are indicative of symmetrical and asymmetrical COO vibrations, while the peak at 1020.07 cm^−1^ represents the C-O-C hemiacetal saccharide units (Figure 3A). No new peaks were found in the FTIR spectrum of the HA- gelatin proteosaccharide; however, an increase in the intensity of the amide II peak was observed at 1542.01 cm^−1^, indicating a molecular interaction in the proteosaccharide (Figure 3C). From the FTIR spectrum of the HA–GEL–GEN, it can be concluded that a semi-IPN is formed in this proteosaccharide combination due to the presence of an amide bond at 1539.72 cm^−1^, indicating the crosslinking of gelatin with genipin, while the characteristic bands of HA at 3285.46 cm^−1^ and 2936.83 cm^−1^, indicative of the carboxylic O-H stretch vibration and CH_2_ groups, respectively, remain (Figure 3D).

### 3.2. Rheological Characterisation of the Crosslinking Reaction

It has been observed that neurons are more prone to thrive when the elastic modulus of a hydrogel is similar to the soft ECM of the brain, whereas astrocytes behave better on stiff substrates [27]. Softer hydrogels induce increased neuronal sprouting as compared to harder hydrogels which lead to neural gliosis [28]. Axonal tips also advance faster on softer hydrogels and appear to retreat from harder areas on a scaffold. Therefore, the elastic modulus required for optimal neuronal growth is <1 kPa [29].

**Figure 4:**
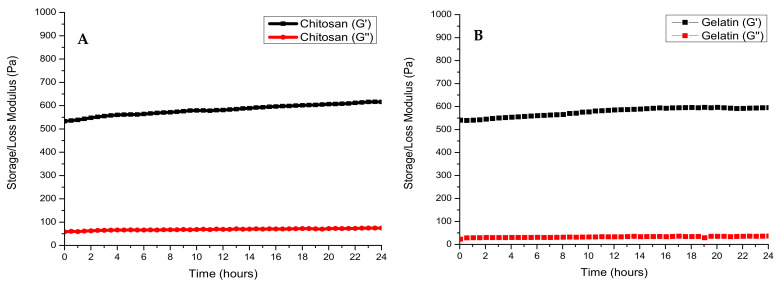

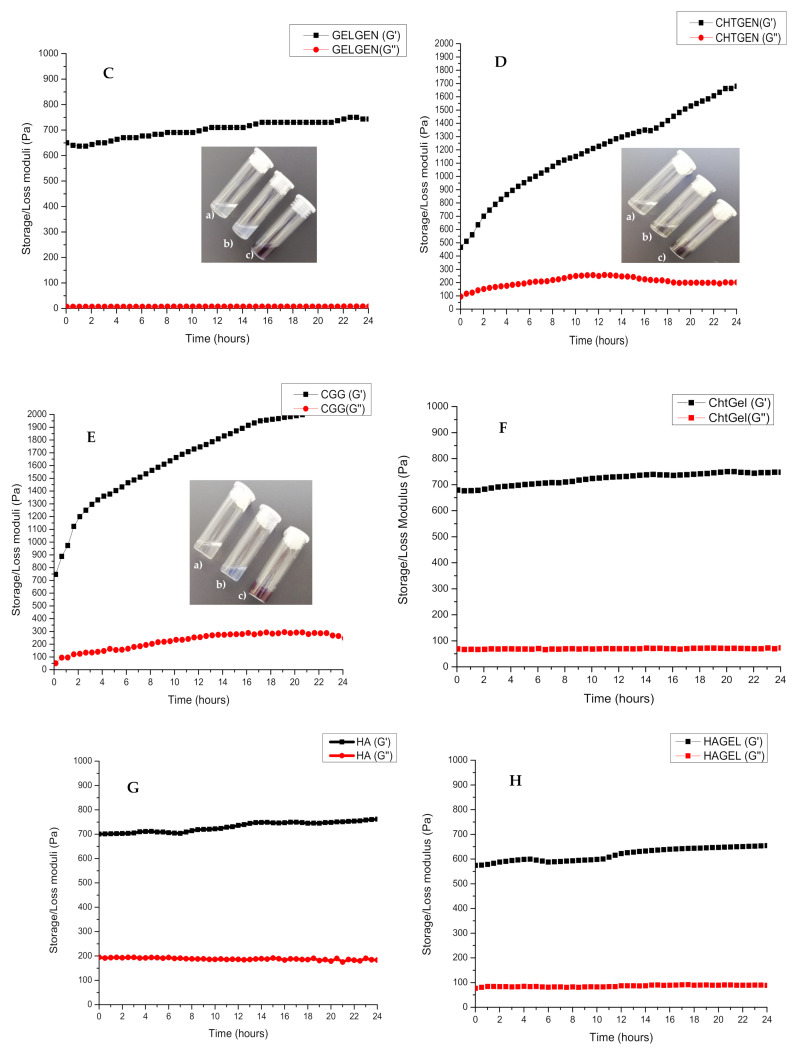

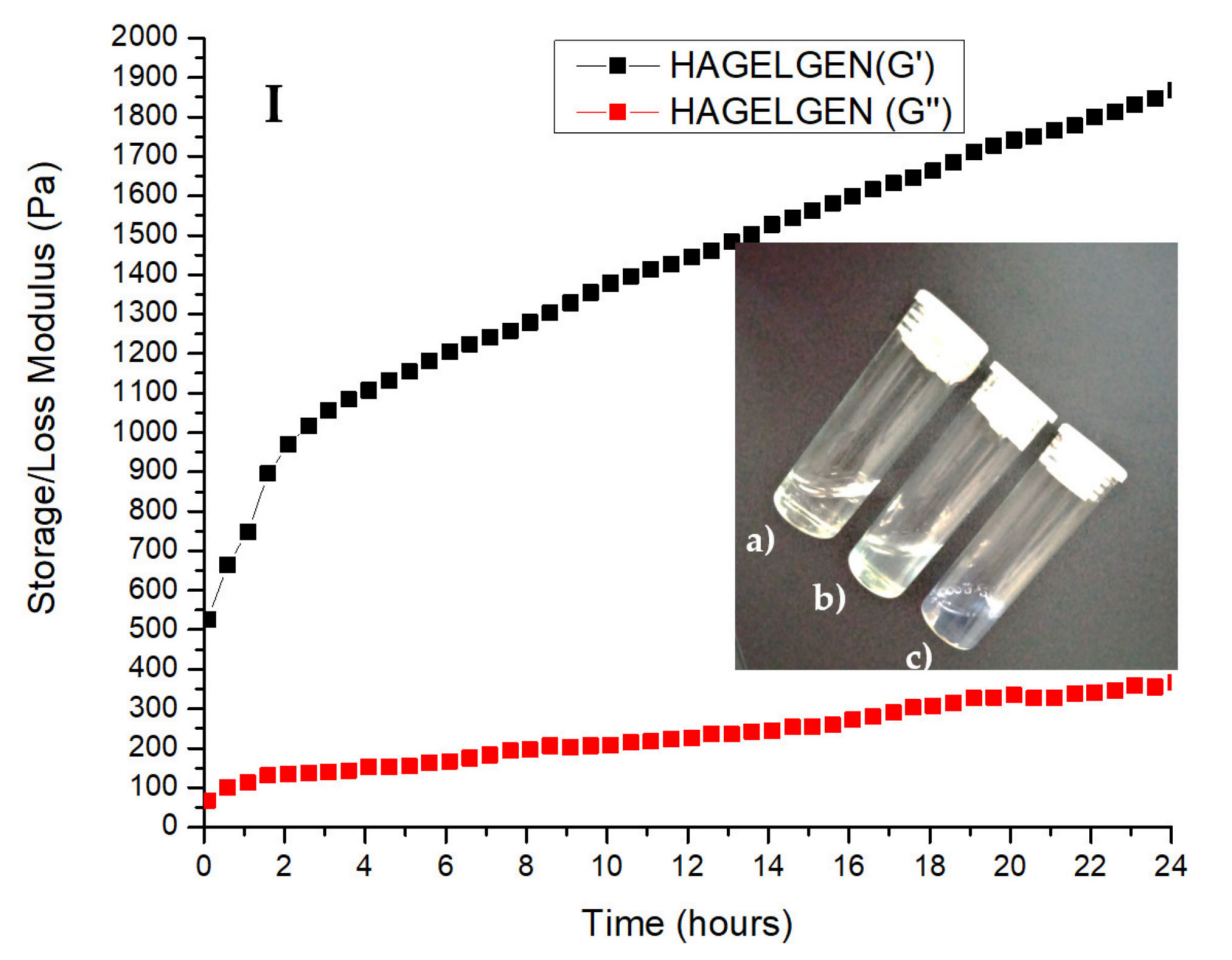
Storage (G′) and loss (G″) modulus of (**A**) chitosan, (**B**) gelatin, (**C**) GELGEN, (**D**) CHTGEN, (**E**) CGG, (**F**) CHTGEL, (**G**) HA, (**H**) HAGEL and (**I**) HAGELGEN. Photographs on each graph show the tilt-bottle test results of each hydrogel at different times of crosslinking: (a) 0 h, (b) 12 h and (c) 24 h.

The rheological behaviour of each polymer and its crosslinked composites were studied over a period of 24 h. Viscoelastic studies of all polymeric composites showed a G′ > G″, which indicated a more solid viscoelastic behaviour. The storage modulus of chitosan had plateaued at 626.2 Pa, while the storage modulus of gelatin had plateaued at 593.8 Pa (Figure 4A, B). This is expected as chitosan is known to exhibit a greater storage modulus than gelatin at a concentration of 2% *w*/*v*. Crosslinking of gelatin with genipin resulted in an increased storage modulus of 743.33 Pa, indicating the formation of amide bonds and greater viscoelastic behaviour (Figure 4C). Crosslinking of chitosan with genipin showed the same effect, with the storage modulus rising to 1678.0 Pa from 465.2 Pa (Figure 4D). The polyelectrolyte complex showed a final G′ of 735 Pa. This is greater than the storage modulus of the pristine polymers, thus indicating a strong electrostatic interaction. The CGG IPN showed the largest storage modulus of 2030.6 Pa due to the formation of amide bonds between the carboxyl group of gelatin and the amino group of chitosan. The final storage modulus of the IPN can be attributed more to the crosslinking of chitosan with genipin resulting in a rigid structural support (Figure 4E,F). The crosslinking of chitosan with genipin occurs at a faster rate than the crosslinking of gelatin which can be deduced from the slope of the graph.

The rheological profile HAGEL network devoid of crosslinker shows an initial storage modulus of 574.2 Pa, less than that of HA due to the incorporation of gelatin at a low concentration and a final storage modulus of 654 Pa (Figure 4G,H). The rheological profile of pristine HA plateaued at 761.67. The water content in the network was increased by combining two aqueous polymers. Both polymers are anionic in nature; therefore, electrostatic interactions are not favourable, and the incorporation of gelatin weakened the network. The rheological curve of the HAGG gel followed the same trend as the full-IPN curve; however, the inclusion of HA resulted in a larger G′ after 24 h. The initial G′ was found to be 526 Pa, and the final G′ was 1868.47 Pa (Figure 4I). All of the rheological profiles indicate that crosslinking with genipin is a timely process and the inclusion of genipin increases the mechanical rigidity of the hydrogels. It can be seen that the effects of crosslinking in each hydrogel can clearly be seen after 4 h. Temperature is also an important factor to consider in crosslinking with genipin, as the reaction proceeds at a faster rate at 40 °C than at room temperature (±25 °C). All of the resulting hydrogels were blue in colour, which was macroscopic confirmation that crosslinking had taken place.

### 3.3. XRD of Each Scaffold

**Figure 5:**
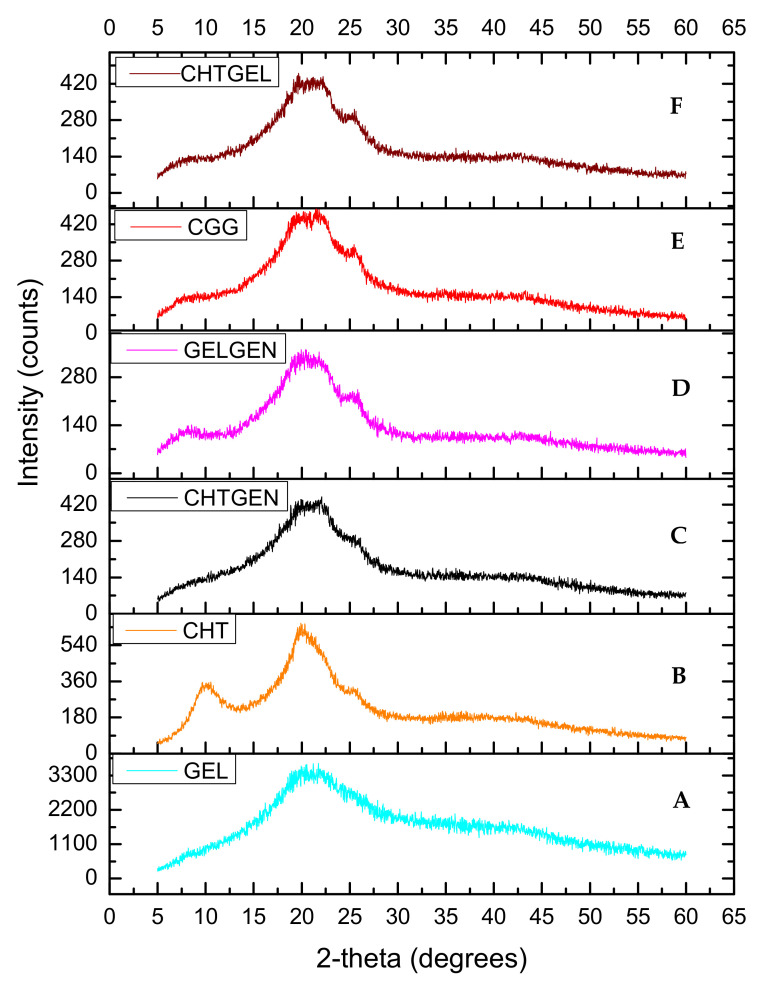
XRD of (**A**) chitosan, (**B**) gelatin, (**C**) CHTGEL, (**D**) CHTGEN, (**E**) GELGEN and (**F**) CGG.

Chitosan and gelatin used in this study displayed amorphous characteristics due to the absence of sharp crystalline peaks and peaks of low intensity (Figure 5A,B). Gelatin typically possesses two peaks at 2θ = 20° and 2θ = 8°. The latter is related to the triple helix content of gelatin. Peaks at 2θ = 10° and 2θ = 20° are typical fingerprints of chitosan powder. When chitosan and gelatin are combined, the peak at 2θ = 8° become flatter, thus indicating that the incorporation of chitosan results in a decreased crystallinity of gelatin [30] (Figure 5C). This can be attributed to hydrogen bonding between the two biopolymers which hinders the triple helix formation of gelatin. When chitosan was crosslinked with genipin, depression of the chitosan peak occurred at 2θ = 20° as genipin reduced the crystallinity of chitosan resulting in an amorphous network (Figure 5D). When gelatin was crosslinked with genipin, gelatin retained its peak at 2θ = 8° with the peak found at 2θ = 25 decreasing in intensity and becoming broader (Figure 5E). The diffractogram of the IPN formed is amorphous in nature and shows a similar pattern to the CHTGEL scaffold; however, the formation of intermolecular crosslinks with genipin resulted in a decrease in crystallinity.

**Figure 6:**
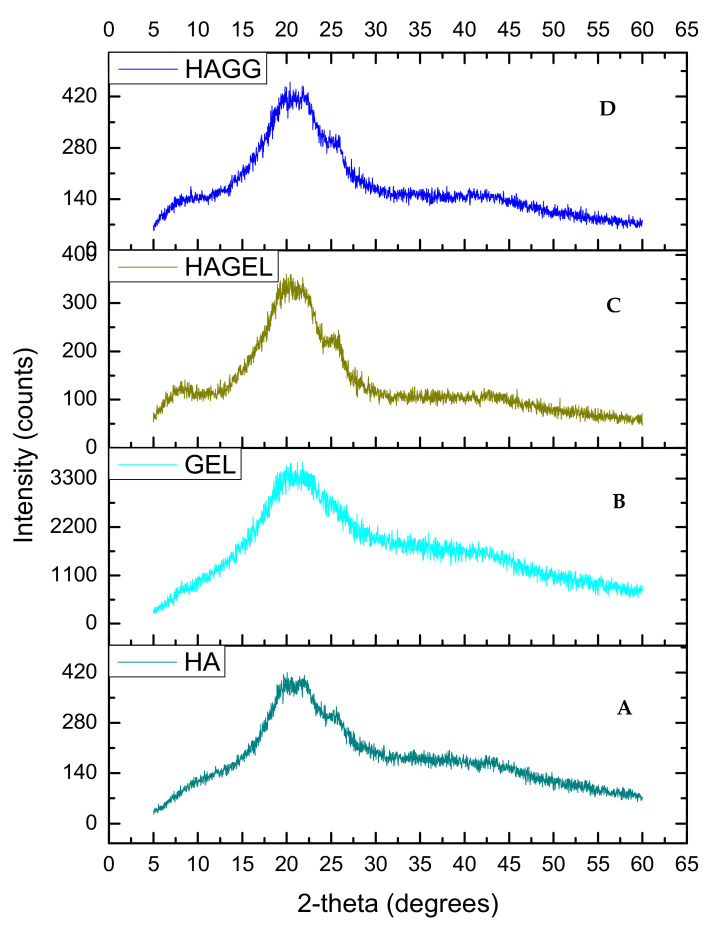
XRD of (**A**) hyaluronic acid, (**B**) gelatin, (**C**) HAGEL and (**D**) HAGELGEN.

The XRD of HA shows two peaks, at 2θ = 20° and a flatter peak at 2θ = 24° (Figure 6A). When gelatin is combined with HA, the same two peaks can be found, indicating that the polymers are miscible and show increased crystallinity due to gelatin incorporation (Figure 6C). The latter peak is flattened when the semi-IPN is formed upon crosslinking with genipin (Figure 6D). Thus the semi-IPN shows a lower crystallinity index than the uncrosslinked network; however, crystallinity is enhanced by incorporating gelatin alone. Crosslinking introduces a defect in the crystalline structure of polymers, and thus, crosslinking results in a reduced crystallinity. The increased density of crosslinking results in a slower crystallisation process and a reduction in the final crystallinity of the networks and rejection of crosslinks into the amorphous phase as shown in Table 1 [31].

It can therefore be concluded that both the full IPN and semi-IPN were amorphous and crosslinking with genipin resulted in an increase in the amorphousness of the networks despite the addition of gelatin, which should have resulted in increased crystallinity [31].

### 3.4. Thermal Stability of Each Scaffold

**Figure 7:**
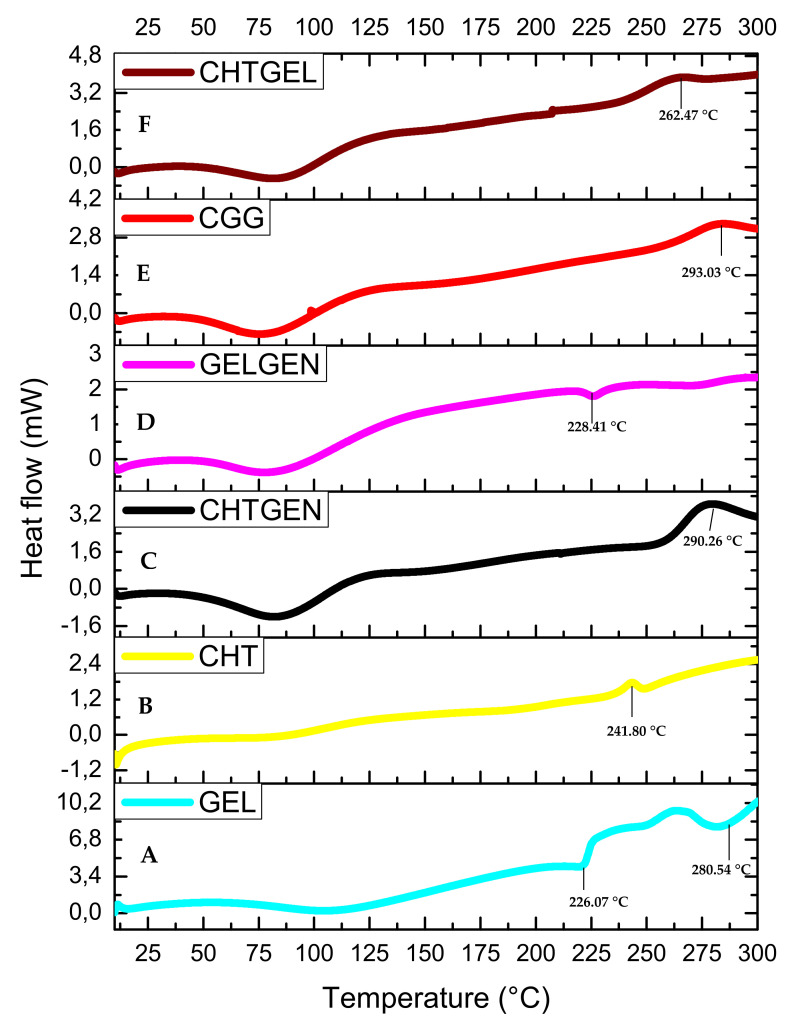
DSC thermograms of (**A**) GEL, (**B**) CHT, (**C**) CHTGEN, (**D**) GELGEN, (**E**) CGG and (**F**) CHTGEL.

The DSC thermogram of gelatin shows three endothermic peaks at 95.04 °C, 226.07 °C and 280.54 °C. The endothermic peak found at 95.04 °C can be attributed to the denaturation of gelatin, i.e., the helix-coil transition of gelatin (Figure 7A). The DSC thermogram of pristine chitosan shows a broad endothermic peak at 99.20 °C, which can be attributed to the loss of water from the sample, which is a non-equilibrium process, and a second thermal event showed by a broad exothermic peak, which can be found at 241.80 °C attributed to the decomposition of glucosamine units in chitosan (Figure 7B). All polymers showed a single endothermic peak between 0 and 125 °C, corresponding to the release of molecular water. When chitosan is crosslinked with genipin, the endothermic peak shifts to 98.31 °C and the exothermic peak shifts to 290.26 °C. Crosslinking results in an increase in thermal stability; however, other researchers agree that the incorporation of genipin leads to an increase in hydrophilicity of the network and therefore results in a lower decomposition temperature due to a lower degree of crosslinking (Figure 7C). When gelatin was crosslinked with genipin, each endothermic peak shifted to higher values, i.e., 86.71 °C, 228.41 °C and 283.19 °C. This can be explained by the reaction of gelatin with genipin, wherein an amide linkage is formed (Figure 7D). The full IPN showed a broad endothermic peak at 87.93 °C and an exothermic peak at 293.03 °C, which suggests greater thermal stability than the proteosaccharide blend, as more energy was required to break the bonds formed due to covalent crosslinking. This, therefore, confirms the formation of a full IPN (Figure 7E). The 50:50 chitosan–gelatin blend showed a broad endothermic peak at 85.15 °C, which can be said to be an intermediate of the Tg of the pristine polymers with a similar peak width to the pristine polymers, thus indicating a single-phase behaviour of the composite and an exothermic peak at 262.47 °C (Figure 7F).

**Figure 8:**
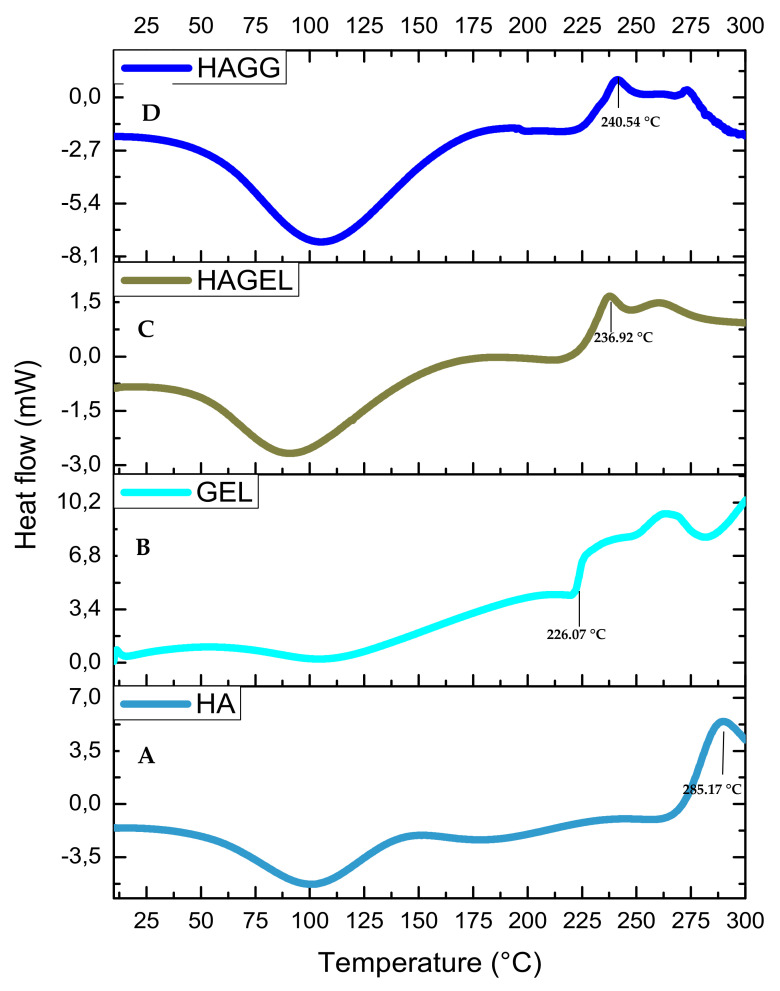
DSC thermogram of (**A**) HA, (**B**) GEL, (**C**) HAGEL and (**D**) HAGELGEN.

The thermogram of HA shows two peaks, an endothermic peak at 80.13 °C corresponding to dehydration of HA and an exothermic peak at 285.17 °C corresponding to the decomposition of the glycosidic bonds between glucosamine and glucuronic acid residues (Figure 8A). The incorporation of gelatin results in an increase in hydrophilicity of the network and a lower decomposition temperature of 236.92 °C (Figure 8C). The semi-IPN is more thermally stable than the HAGEL blend and exhibits a higher decomposition temperature at 240.54 °C due to the formation of amide bonds in gelatin after reacting with genipin (Figure 8D). Therefore, it can be concluded that the combination of the two hydrophilic polymers results in lower thermal stability, which is slightly improved by crosslinking one of the polymers, that being gelatin in this study. Thermal stability would be significantly improved by modifying HA, using thiolation or methacrylation [32,33].

### 3.5. Swelling Potential of Each Scaffold

**Figure 9:**
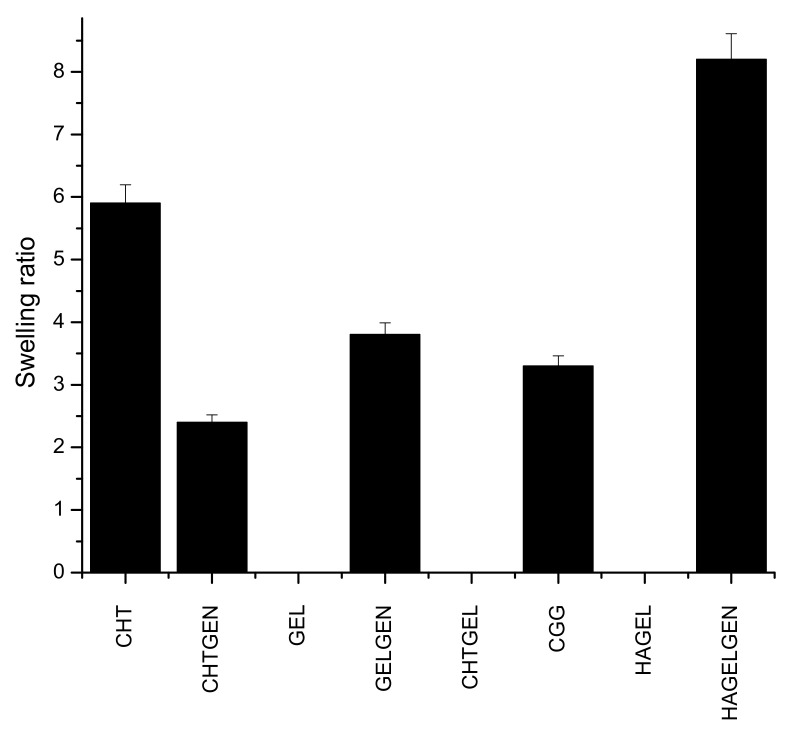
Bar graph showing the swelling ratio of each formulation (n = 3).

Hydrogel scaffolds are preferred due to their ability to swell and retain water. Many factors such as crosslinking density, crystallinity and porosity influence the swelling of a hydrogel scaffold [34]. This has implications on degradation and permeability. Swelling enhances biomimicry in the context of the CNS due to the increased mobility of metabolites and nutrients in the scaffold. The brain is composed of ~80% of water; therefore, the scaffold must be able to swell and retain its architectural morphology instead of dissolving in the fluid rich environment [35]. Gelatin and HA undergo dissolution in the absence of a crosslinker. Chitosan undergoes a certain level of swelling in the absence of genipin before undergoing dissolution due to the greater mechanical strength of the polymer, which supports the reason for its employment in this study. Swelling is inversely proportional to the degree of crosslinking, which explains why crosslinked networks swell less than their uncrosslinked counterparts as shown in Figure 9 [36]. The swelling ratio of CHTGEN is increased by the incorporation of genipin due to Chitosan alone containing hydrophobic residues and genipin increasing the hydrophilicity of the network. The full IPN showed a higher swelling ratio than GELGEN, but a lower swelling ratio than CHTGEL and CHTGEN, thus proving the effects of crosslinking. The HAGEL network dissolved in less than 1 h, while the HAGG network showed a swelling ratio of 8.31, which is close to that of GELGEN, indicating that the semi-IPN was able to retain its architecture instead of dissolving due to the crosslinking of the gelatin component. High swelling ratios of scaffolds can lead to tissue compression and subsequent damage; therefore, crosslinking was employed to reduce the swelling ratio and to increase the mechanical strength of the scaffold [37] (Figure 9).

### 3.6. Morphological Analysis of Proteosaccharide Scaffolds

**Figure 10:**
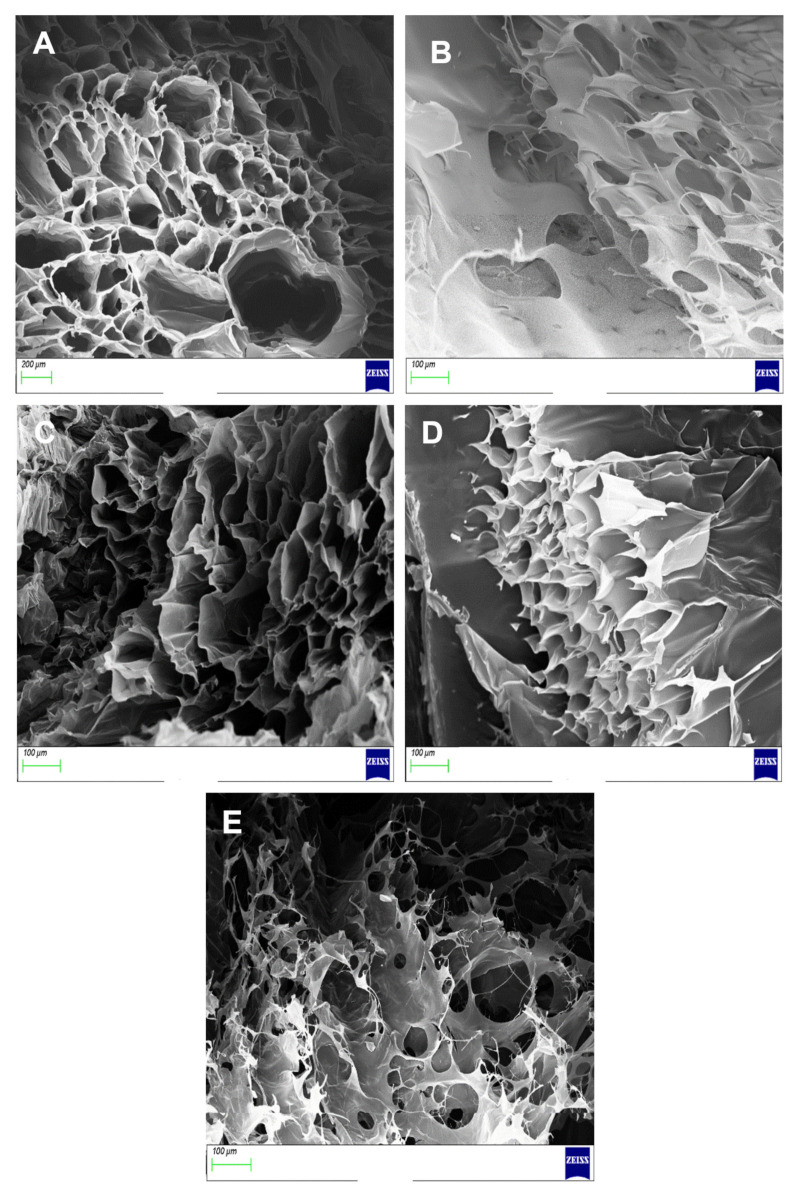
SEM images of (**A**) CHTGEN, (**B**) GELGEN, (**C**) CGG, (**D**) HAGEL and (**E**) HAGELGEN.

Transverse cross-sections of each hydrogel scaffold were used to calculate the average diameter of each pore and porosity shown in Table 2. The SEM micrograph of CHTGEN showed a microporous structure with well-defined and carefully delineated pores. The pores were geometrically round, with an average diameter of the pores being 148.920 μm ± 6.335 (Figure 10A). The SEM micrograph of GELGEN revealed very thin walls and a very fibrous structure. This can be attributed to the low concentration of gelatin used in this study, therefore resulting in low mechanical integrity (Figure 10B). The average pore size was calculated to be 65.298 ± 9.936 μm, which was much smaller than the CHTGEN scaffold. The CGG network showed a tissue-paper like structure but showed better-defined pore walls, which was sourced from CHTGEN crosslinking, with an average pore size of 72.789 ± 16.85 µm. Pores were more closely packed, with smaller pore diameters and thicker walls than the GELGEN scaffold (Figure 10C). The HAGELGEN scaffold showed a microporous structure, which was difficult to observe on a transverse cross-section due to the linear arrangement of HA fibres. The average diameter of the pores was calculated at 84.289 ± 7.65 μm, which is smaller than that of the uncrosslinked blend (Figure 10D,E). The presence of pores is imperative to the architecture of the scaffold. Both scaffolds showed the presence of multiple pores. The porosity was calculated using the SEM images, and the CGG scaffold was less porous (71.335%) than the GELGEN scaffold (76.985%) but more porous than the CHTGEN scaffold (52.791%). The HAGELGEN scaffold showed a porosity of 73.614%. A porous scaffold is crucial for the diffusion of oxygen and nutrients in the scaffold. The CHTGEN scaffold was the least porous due to the higher crosslinking density of the scaffold. Genipin was able to crosslink chitosan to a greater effect than it was able to crosslink gelatin. The GELGEN scaffold showed a high porosity due to the low concentration of gelatin employed, as well as the low crosslinking density. The HAGELGEN scaffold also showed a high porosity due to the largely hydrophilic network formed and the freeze-drying process resulting in a loss of some molecular water. Other researchers have used scaffolds with an 85% porosity for neural tissue engineering [38,39]. Both the IPN and semi-IPN showed comparable porosities and would be compatible for the migration of cells in vitro while maintaining their structural integrity.

### 3.7. Density of Scaffolds

Density has a direct implication on the swelling potential and can be directly related to the crosslinking density of the polymeric networks. The bulk density of each scaffold was calculated according to Equation (3) and is shown in Table 3. The semi-IPN was surprisingly the densest network. This could be due to the high molecular weight of HA, which resulted in a heavier scaffold, as compared to the full IPN. The full IPN was denser than the CHTGEN and GELGEN scaffolds, therefore proving that crosslinking of both polymers results in a dense hybrid scaffold, which swells less and should be more resistant to degradation. There were more amide bonds formed in the full IPN encompassing both chitosan and gelatin.

### 3.8. In Vitro Degradation of Proteosaccharide Scafffolds

**Figure 11:**
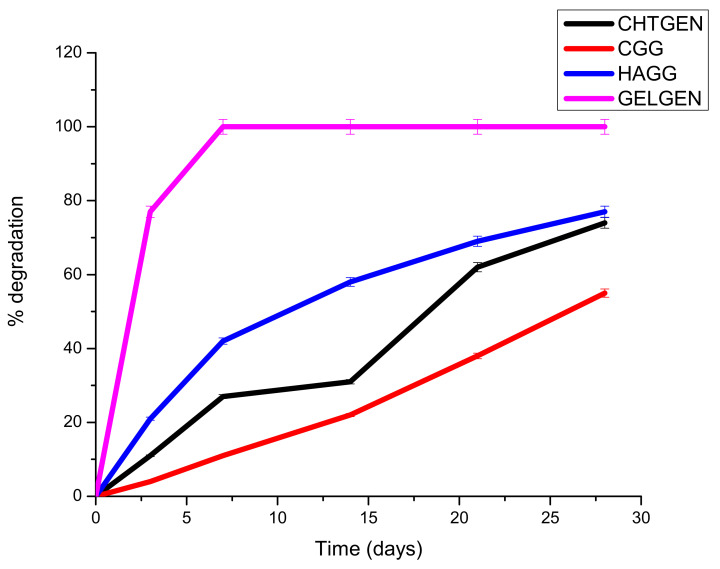
Degradation of genipin-crosslinked scaffolds undertaken over 30 days in the presence of PBS (n = 3).

A scaffold must be mechanically robust and degrade constantly into non-toxic by-products. Biodegradability prevents the unnecessary process of removal of the scaffold, which could cause secondary injury. Surface erosion is favoured over bulk erosion to allow the scaffold to maintain its stable architecture and not collapse in vivo. Ideally, a scaffold should degrade at a rate that matches that of neogeneration [35]. A scaffold that degrades too rapidly results in the accumulation of degradation components, resulting in a hypertonic and stressful environment for cells. Furthermore, cells are not given enough time to establish a niche within the scaffold to drive neural regeneration. The hydrophilicity and hydrophobicity of the system are also pertinent to its degradation behaviour. A largely hydrophilic system will absorb more water and undergo rapid bulk degradation; therefore, polymer ratios must be carefully selected. The gelatin-genipin scaffold degraded too rapidly, with greater than 78% of its weight lost on day 3 only. This can be explained by the hydrophilic nature of gelatin and the low degree of crosslinking. For this reason, gelatin needed to be combined with other components, i.e., chitosan and HA. The semi-IPN showed a faster degradation rate than the full IPN. This is due to the presence of HA, which is a hydrophilic polymer. HA encouraged swelling and subsequent degradation; however, the scaffold was able to retain its shape for the full 28 days. The full IPN showed a slower degradation rate, with only 47% degraded at day 28. This was due to the formation of amide bonds between the amine of chitosan and the carboxyl group of gelatin. This led to greater stability and resistance to enzymatic degradation (Figure 11). The degradation behaviour can be further tuned by adjusting the ratio of polysaccharide to gelatin, which would greatly improve the rigidity of the scaffold; however, careful consideration must be given to the mechanical strain that a hard scaffold can exert on neural tissue, which could lead to secondary damage. Furthermore, the use of more crosslinkers can prove to be toxic to cells.

### 3.9. Drug Release of Dexamethasone-21-Phosphate from the IPN and Semi-IPN Hydrogel Scaffolds

Dexamethasone-21-phosphate is a synthetic glucocorticoid steroid with anti-inflammatory activity. DEX can therefore play an important role in TBIs due to its ability to bind to astrocyte glucocorticoid receptors and prevent the proliferation of astrocytes [40,41]. Furthermore, DEX can lower the production of cytokines such as IL-1β, INF-γ and TNF-α [40]. The use of other corticosteroids such as high-dose methylprednisolone is not permitted for use in patients with TBIs to improve their therapeutic outcomes according to the Brain Trauma Foundation; however, a study conducted by Moll et al. showed the beneficial effect of DEX in patients with brain contusions [42]. Another double-blinded study showed that DEX administered in high doses reduced mortality and improved neurological outcomes in patients with head injuries [43]. Therefore, the release of DEX from hydrogel scaffolds was tested. A calibration curve was constructed, using concentrations ranging from 5 µg/mL to 25 μg/mL, and the linear regression coefficient (R2) was determined to be 0.998. The equation of the graph was used to calculate the concentration of drug released at each interval.

**Figure 12:**
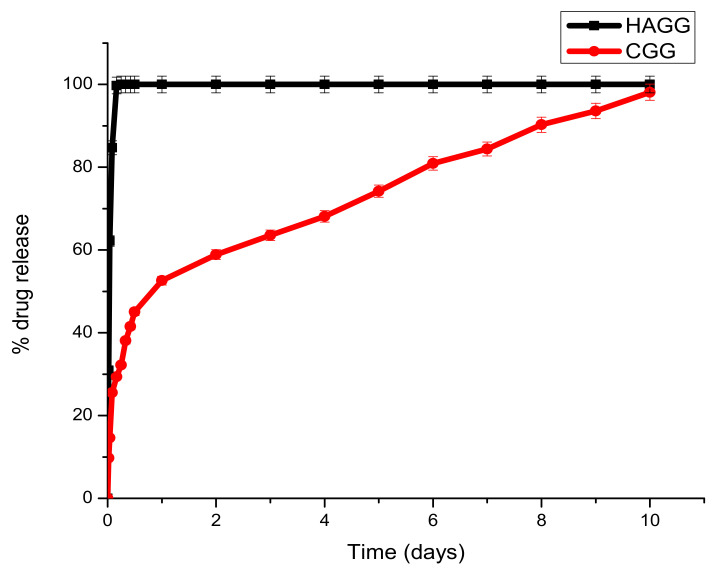
Graph depicting the release of DEX-21-phosphate from the full IPN and semi-IPN (n = 3) SD < 2.

Dex-21-phosphate is a highly soluble phosphate salt of DEX, a systemic glucocorticoid. Due to its high water solubility, the delivery system needs to be densely crosslinked to prolong its release. From the drug release profiles, it can be seen that the semi-IPN does not support the sustained release of DEX-21-phosphate, due to the low crosslinking density of gelatin by genipin and the low concentration of gelatin used, which does not allow for the formation of a strong hydrogel network. A burst release of DEX-21-phosphate was seen in both the IPN and semi-IPN; however, the semi-IPN showed a 100% release in 6 h (Figure 12).

Under clinical circumstances, the fast release of DEX-21-phosphate could be beneficial in reducing local inflammation post-traumatic brain injury and in the prevention of inflammatory cells such as astrocytes and macrophages, thus preventing glial scar formation. The full IPN showed two phases of drug release. A burst release was seen in the first 24 h, with 52.61% of the drug being released. A second phase observed from day 2 to day 7 was due to the decay of the polymeric matrix and the release of entrapped drugs according to linear kinetics. This would be beneficial in the clinical setting by preventing local oedema surrounding the lesion and allowing the scaffold to swell and serve as a surrogate for tissue repair. Burst releases occur due to drug molecules that are trapped on the surface of the scaffold being released, and the sustained release can be attributed to drug molecules entrapped within the core of the matrix.

### 3.10. Cytotoxicity Analysis of Proteosaccharide Hydrogels (IPN and Semi-IPN)

**Figure 13:**
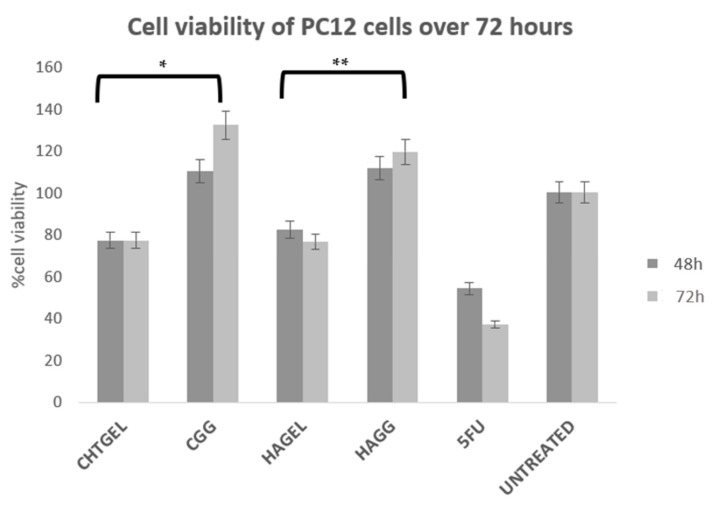
Cytotoxicity analysis of crosslinked and uncrosslinked formulations, performed on PC12 cells for 72 h, using an XTT cell viability assay (SD < 0.2) (n = 3). 5-FU (5-Fluorouracil, 10 µg/mL) was used as the positive control (n = 3) (* *p* < 0.0001, ** *p* = 0.0011).

Cell viability was calculated and expressed as a percentage and compared to the untreated wells. Cell viability of above 80% is considered non-cytotoxic, 60–80% is considered weak toxicity, 40–60% is considered moderate toxicity and <40% is considered severely toxic according to ISO 10993-5. Cell viability (XTT) assays revealed that the blends of chitosan and gelatin, as well as the crosslinked hydrogels, showed a cell viability of greater than 70%. Chitosan, gelatin and HA are considered to be non-cytotoxic and biocompatible; however, at 72 h, more cell death was noticed due to the system not being able to provide adequate mechanical strength to support cell growth. The CHTGEL and HAGEL blends showed an average cell viability of 77.201% and 76.527%. The effect of crosslinking on cell behaviour can be seen in the graph. The crosslinked hydrogels showed greater viability than the non-crosslinked hydrogels, with the full IPN showing viability of 132.24% and the semi-IPN showing viability of 119.56%. It can be deduced that crosslinking enhanced the stiffness of the hydrogel and cellular activity. PC12 cells have a doubling time of 48 h; therefore, a 24 h assay was not carried out. Gelatin contains the RDG sequence, which supports cell attachment and proliferation. Genipin has proven to have neuritogenic activity in PC12 cells and neuroprotective effects on β-amyloid peptide-treated primary hippocampal neurons of rats that express nNOS proteins [44]. Furthermore, the crosslinked hydrogels showed cell viability greater than that of the control. Genipin-crosslinked hydrogels can be applied in TBIs due to their neuroprotective effect, and it could prevent further secondary damage. This can be attributed to its architectural similarity to the ECM, which permits cell attachment and proliferation. The XTT results, therefore, show that both the IPN and semi-IPN were neurocompatible and show promising potential in neural regeneration (Figure 13).

**Figure 14:**
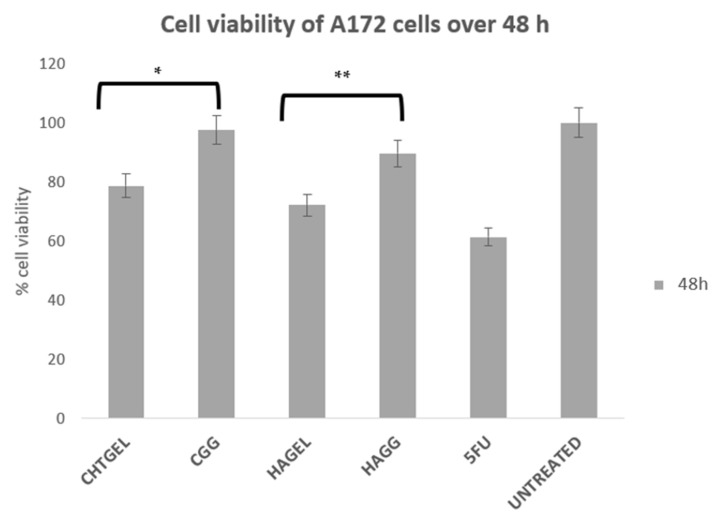
Cytotoxicity analysis of the crosslinked and uncrosslinked formulations, carried out on A172 cells over 48 h using an XTT cell viability assay (SD < 0.3) (n = 3). 5-FU (5-Fluorouracil, 10 µg/mL) was used as the positive control (* *p* = 0.0009, ** *p* = 0.0005).

A172 cells proliferate more rapidly than PC12 cells. This is notably due to their nature as human glioblastoma cells, with their doubling time ranging from 40 to 44 h. Therefore, for the purpose of this study, an XTT study was performed at 48 h. At 72 h, the rapid rate of proliferation and the limited growth media would result in apoptosis. The XTT assay of A172 cells showed results that are similar to the results obtained from the PC12 cell line (Figure 14). The uncrosslinked formulations showed a lower cell viability of 78.663% for CHTGEL and 72.136% for HAGEL. The IPN and semi-IPN showed cell viability of 97.52% and 89.72%, respectively. The apoptosis caused was due to the incorporation of genipin, which has antiproliferative effects on glioblastoma cells. Ahani and co-workers (2018) demonstrated that genipin had an inhibitory effect on uncoupling protein 2 (UCP2), which causes proton leaks through the inner mitochondrial membrane and uncouples oxidative phosphorylation from ATP synthesis and negatively regulates the production of reactive oxygen species (ROS) in the mitochondria. Genipin induces apoptosis through the UCP2 pathway and the induction of intracellular ROS [45].

### 3.11. Migration of A172 Cells in Response to the Full IPN and Semi-IPN

Migration studies were performed on A172 cells for a period of 48 h due to their faster doubling time (44 h) compared to PC12 cells (50–60 h) and easier attainability of a cell monolayer, through which a clear-cut lesion could be made. Cells are known to migrate due to their response to biophysical, topographical and chemical cues provided by the hydrogels. The migration of A172 cells is evident from the images below.

**Figure 15:**
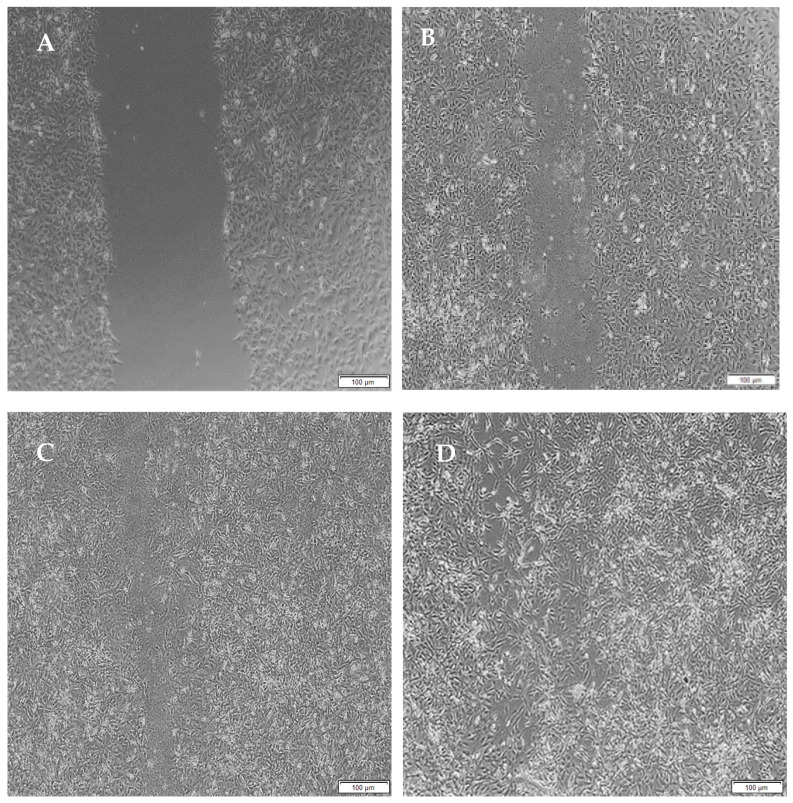
Micrographs of the migration of A172 cells from a wound-healing assay of the full IPN at (**A**) 0 h, (**B**) 8 h, (**C**) 24 h and (**D**) 48 h. Images were captured using the 4× objective and the phase contrast feature of an inverted light microscope. The red arrows indicate actively migrating cells at 8 h.

**Figure 16:**
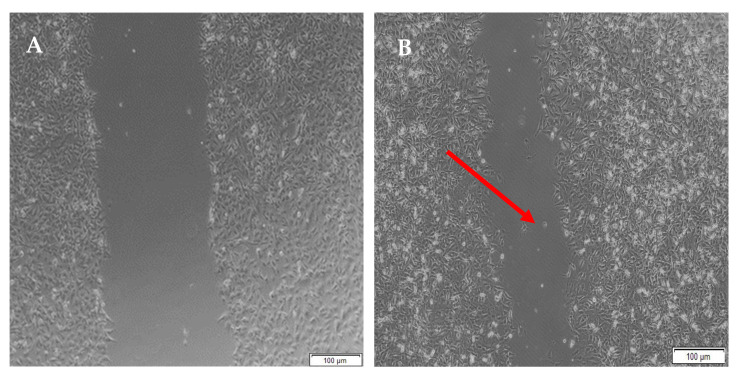

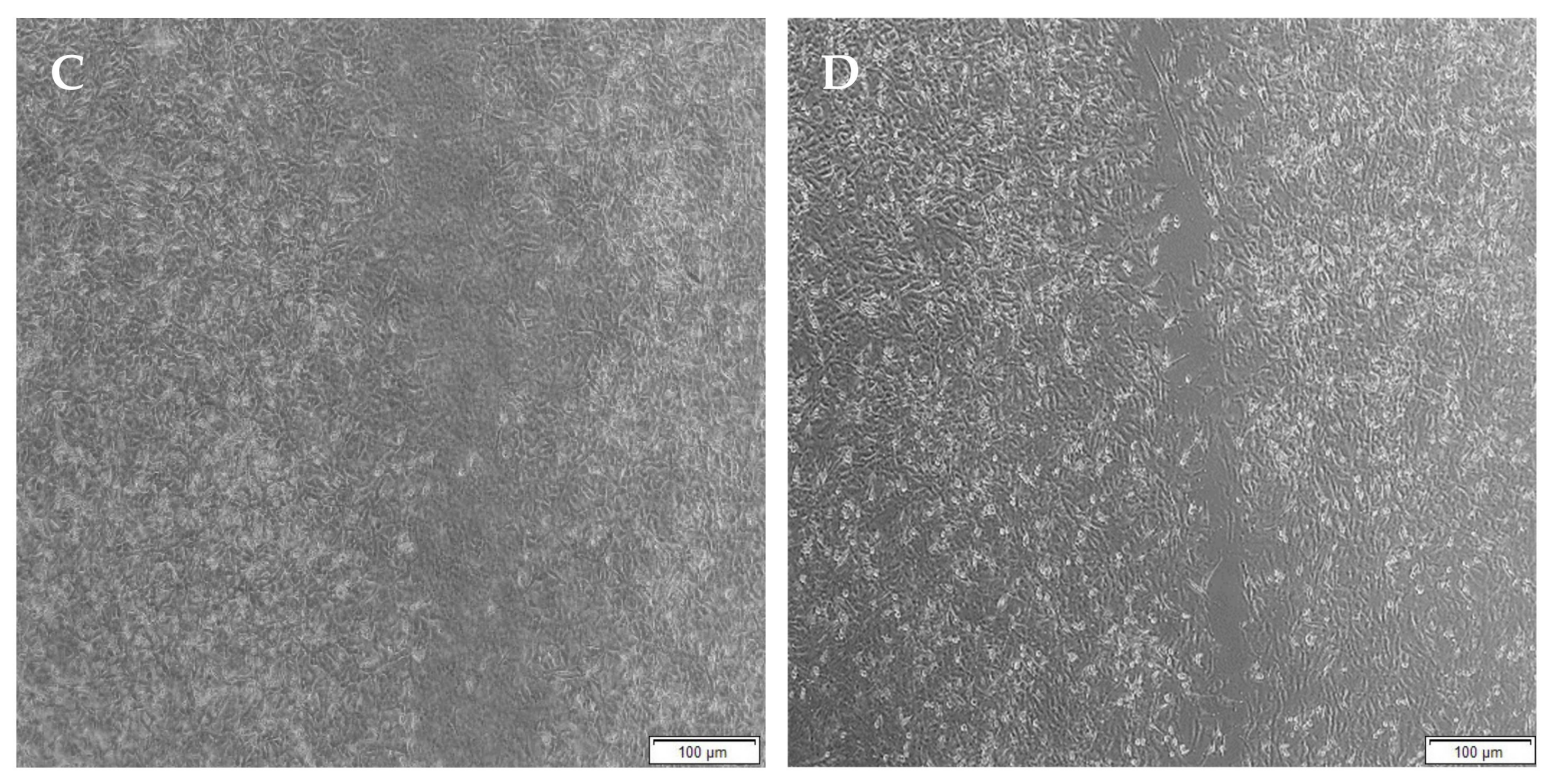
Micrographs of the migration of A172 cells from a wound-healing assay of the semi- IPN at (**A**) 0 h, (**B**) 8 h, (**C**) 24 h, and (**D**) 48 h. Images were captured using the 4× objective and the phase contrast feature of an inverted light microscope. The red arrows indicate actively migrating cells at 8 h.

**Figure 17:**
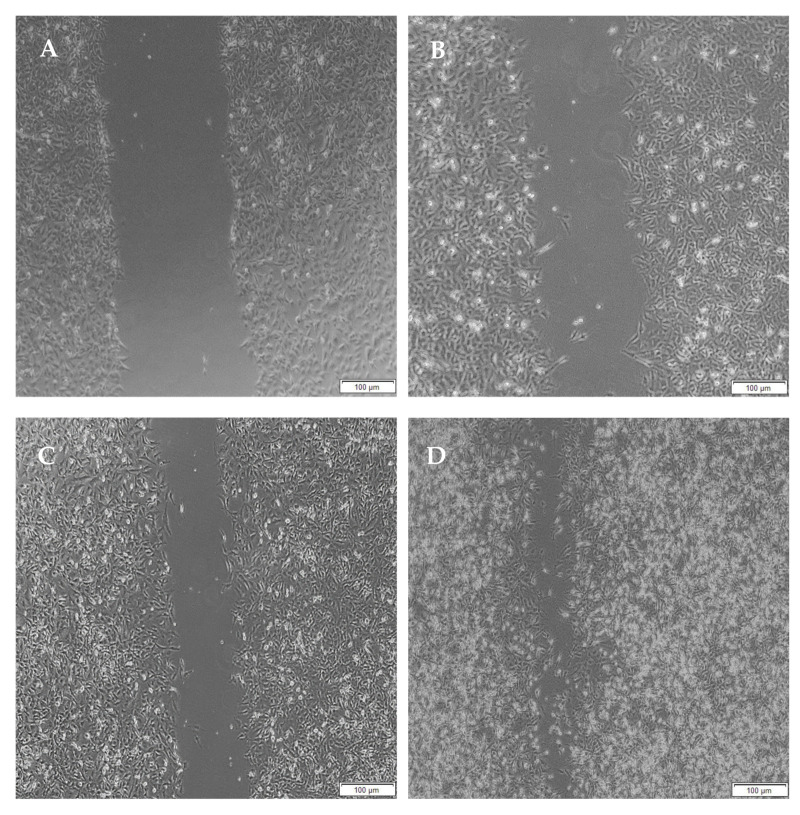
Micrographs of the migration of A172 cells from a wound-healing assay of the untreated at (**A**) 0 h, (**B**) 8 h, (**C**) 24 h and (**D**) 48 h. Images were captured using the 4× objective and the phase contrast feature of an inverted light microscope. The red arrows indicate actively migrating cells at 8 h.

**Figure 18:**
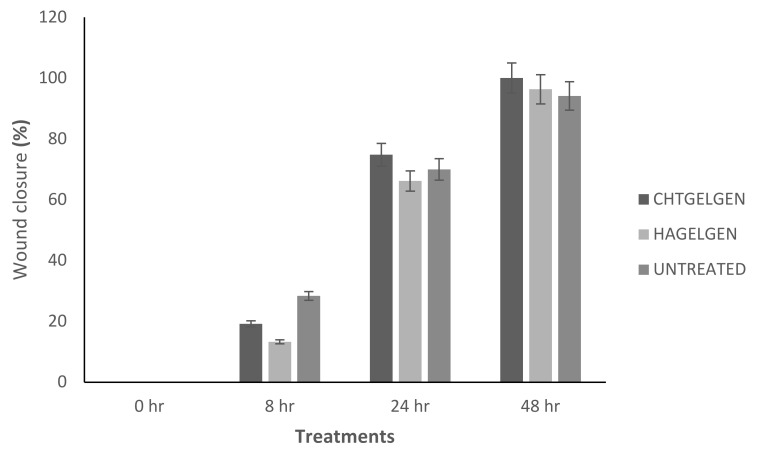
Bar graph showing the percentage wound closure of A172 cells at each interval calculated using NIH ImageJ wound healing tool (n = 3) (SD < 2.52).

When the lesion was made (t = 0), a clear gap can be seen between the two sides of each well. At 8 h, the semi-IPN showed an average wound closure of 13.27%, which was comparable to the full IPN, which showed closure of 19.19% (Figure 15 and Figure 16B). At 24 h, the wound closure of all the treated wells showed a wound closure of greater than 60%. At 24 h, each treatment was given enough time to interact with the plated cells after being diluted in growth media. The CGG hydrogel showed a wound closure of 74.79%, which exceeded that of the HAGELGEN hydrogel (66.14%) (Figure 15 and Figure 16C). The full IPN hydrogel showed greater mechanical stability and thereby encouraged the migration of A172 cells to fill the lesion. In the gelatin containing hydrogels, the RDG peptide served as a hapotactical cue to direct cell migration. At 48 h, all of the lesions were closed, with the HAGG wound being 90.521% closed and the CGG wound being 100% closed (Figure 14 and Figure 15D). The untreated wound was only 94.12% closed (Figure 17D). This showed that the two hydrogels demonstrated better wound healing properties than the untreated formulation. The full IPN showed a faster rate of migration than the semi-IPN. However, both hydrogels are able to support cell migration and are neurocompatible. A large amount of apoptosis can be seen in the images corresponding to the 48 h time points. This was due to media not being replaced and the rapid doubling time, which resulted in some cell death. Additionally, a high seeding density was used to create a clear, linear lesion. The images of the 8 h and 24 h time points are turbid due to the presence of the hydrogel, which could not be washed, to monitor cell migration. The average wound closure for each hydrogel has been represented in Figure 18, with each value calculated from the ImageJ wound-healing plugin (Figure 18). 

## 4. Conclusions

From the research conducted, it can be concluded that both chitosan and HA can be combined with a bioactive protein, such as gelatin and thereafter crosslinked with the non-toxic crosslinker, genipin, to form hydrogel scaffolds for potential neural regeneration. The main advantage of the inclusion of these polymers is the increased mechanical properties that they add to the scaffold. The CGG scaffold can prove as a drug delivery system for hydrophilic corticosteroids, like DEX-21-phosphate. The effect of DEX-21-phosphate must still be tested in vitro, which was not carried out in this study. Further modification of HA using methacrylation or thiolation can be carried out to improve the strength and prolong the release of DEX-21-phosphate.

## Figures and Tables

**Table 1:** Crystallinity index of each scaffold calculated from XRD (n = 3).

Scaffold	Ci	Scaffold	Ci
Chitosan	0.61821 ± 0.0599	Hyaluronic acid	0.43432 ± 0.0152
Gelatin	0.51431 ± 0.0212	Hyaluronic acid–Gelatin	0.51289 ± 0.0178
Chitosan–Gelatin	0.62533 ± 0.0374	Hyaluronic acid Gelatin–Genipin	0.49226 ± 0.0210
Chitosan–Genipin	0.46459 ± 0.0165	Chitosan–Gelatin–Genipin	0.50380 ± 0.0410
Gelatin–Genipin	0.52687 ± 0.0138		

**Table 2:** Calculated average pore diameters and porosity of each scaffold (n = 10).

Scaffold	Average Pore Diameter (µm)	Porosity (%)	Scaffold	Average Pore Diameter (µm)	Porosity (%)
CHTGEN	148.920 μm ± 6.335 µm	52.791%	HAGEL	107.54 μm ± 13.52 µm	73.614%
GELGEN	65.298 μm ± 9.936 µm	76.985%	HAGELGEN	84.289 μm ± 7.658 μm	46.257%
CGG	72.789 μm ± 16.85 µm	71.335%			

**Table 3:** Density of the pristine polymers and their crosslinked scaffolds (n = 3).

Combination	Density (mg/mm^3^)	Combination	Density (mg/mm^3^)
CHT	0.048 ± 0.002	CHTGEN	0.1345 ± 0.037
GEL	0.0323 ± 0.016	GELGEN	0.0572 ± 0.028
HA	0.0457 ± 0.021	HAGG	0.1967 ± 0.059
CHTGEL	0.0573 ± 0.013	CGG	0.1476 ± 0.065
HAGEL	0.0276 ± 0.008		

## Data Availability

The data presented in this study are available on request from the corresponding author.
